# The Prevalence of Syphilis, HTLV I/II, and Malaria Among Blood Donors at the Riyadh Regional Laboratory: A Retrospective Study from Saudi Arabia

**DOI:** 10.3390/jcm15187058

**Published:** 2026-09-11

**Authors:** Nouf Shawqi Alwaseah, Abdulwahab Binjomah, Rimah Abdullah Saleem, Muhammad Raihan Sajid, Hani Tamim, Maria Imtiaz, Ashna Majid

**Affiliations:** 1 College of Medicine, Alfaisal University, Riyadh 11533, Saudi Arabia; 2 Bacterial Diseases and Special Pathogens Department, Public Health Authority, Riyadh 13351, Saudi Arabia; 3 Department of Biochemistry and Molecular Medicine, College of Medicine, Alfaisal University, Riyadh 11533, Saudi Arabia; 4 Department of Pathology, College of Medicine, Alfaisal University, Riyadh 11533, Saudi Arabia; 5 Department of Internal Medicine, Clinical Research Institute, American University of Beirut Medical Center, Beirut 1107 2020, Lebanon

**Keywords:** transfusion-transmissible infections, syphilis, HTLV I/II, malaria, blood donors, Saudi Arabia, public health, screening

## Abstract

**Background:** Transfusion-transmitted infections (TTIs) continue to threaten the safety of blood supply systems worldwide. This study aimed to estimate the prevalence of malaria, HTLV I/II, and syphilis among blood donors at the Riyadh Regional Laboratory and Blood Bank from 2018 to 2022, and to assess associations with gender, age, nationality, and the ABO/RhD blood group. **Methods:** We conducted a retrospective analysis of 217,543 blood donations. Demographic and serological records were extracted from the blood bank’s electronic database and analyzed in SPSS v28. Chi-square, Fisher’s exact tests and multivariate logistic regression were used as appropriate, with significance set at *p* < 0.05. **Results:** Male donors accounted for 97% of the cohort, and 52% were non-Saudi nationals. Malaria was rare (0.03%; *n* = 58); all 58 cases were detected in 2018, and none were recorded in subsequent years. HTLV I/II antibodies were observed in 464 donors (0.21%), with the highest annual share in 2019 (39.7%). Syphilis was the most common TTI, identified in 1143 donors (0.52%), and showed a significant increasing trend over the study period (Cochran–Armitage test *p* < 0.001; Poisson regression IRR 1.21, 95% CI 1.15–1.28, *p* < 0.001). In multivariable logistic regression, non-Saudi nationality was independently associated with syphilis positivity (aOR 1.48, 95% CI 1.31–1.67), and ages 46–65 was associated with higher odds of all three TTIs. ABO and RhD blood groups were not significantly associated with TTI positivity in univariate or multivariable analyses. **Conclusions:** Current screening practices appear to maintain low TTI prevalence in the central region, but the rising syphilis trend and the clustering of malaria positivity among non-Saudi donors point to the need for closer surveillance and targeted donor counseling. The study also highlights the need for more granular demographic data collection, including specific country of origin and duration of residence, to enable more effective risk stratification and intervention targeting.

## 1. Introduction

Blood transfusion remains an essential part of clinical care. It is used to manage trauma, major surgery, obstetric hemorrhage, hematologic disease, and many other conditions in which adequate oxygen-carrying capacity must be restored quickly [1]. More than 100 million units of blood are collected worldwide each year [2]. In Saudi Arabia, approximately 350,000 blood donations were collected annually over the previous decade (2010–2020) [3]. Although blood transfusion is generally safe, there remains a potential risk of transmitting infections from donor to recipient when the screening process fails [2,4].

Saudi Arabia is one of 166 countries that screen all blood donations as part of national policy [1]. Eligible blood donors in Saudi Arabia must be between 18 and 65 years of age, be in good health, weigh more than 50 kg, and have a hemoglobin level of at least 12.5 g/dL in females and 13.5 g/dL in males [5]. The Saudi Ministry of Health (MOH) requires testing for hepatitis B surface antigen (HBsAg), hepatitis B core antibodies (anti-HBc), hepatitis C virus antibodies (anti-HCV), human immunodeficiency virus antibodies (anti-HIV-1/2), human T-cell lymphotropic virus-I and -II antibodies (anti-HTLV-I/II), *Treponema pallidum* antibodies, and malaria, along with nucleic acid testing (NAT) for HIV-1/2, HCV, and HBV [5]. To be eligible for blood donation, donors must also have normal vital signs, including blood pressure within the acceptable range (systolic 120–129 mmHg and diastolic 80–89 mmHg), a pulse rate of 60–100 beats/min, and a body temperature below 37 °C [6].

Transfusion-transmitted infections (TTIs) remain a public health concern, especially in countries with diverse donor populations and varying patterns of disease endemicity. Since the identification of the ABO blood group, interest in how blood groups may influence infectious diseases has grown [7]. Because blood types are genetically determined traits with documented polymorphic expression among individuals and populations, they are often studied in epidemiological research [8]. Blood group antigens may also act as decoy receptors, preventing pathogens from attaching to their target tissues. Furthermore, microorganisms can elicit antibodies against blood group antigens, such as those of the ABO system. In the context of certain bacterial infections and enveloped viruses that express ABO-active antigens, ABO antibodies may be considered a part of the innate immune system [7].

Three clinically significant transfusion-transmitted infections—syphilis, HTLV-I/II infection, and malaria—are particularly relevant to the Saudi context. Syphilis is a chronic multistage disease caused by the spirochete bacterium *Treponema pallidum* subspecies pallidum [9]. The pathogenesis and antigenic variation in *T. pallidum* are areas of active research, with studies investigating genetic mechanisms such as homopolymeric repeats that may regulate the expression of outer membrane proteins involved in host–pathogen interactions [10]. The infection is primarily transmitted through sexual contact with infectious lesions, although congenital and transfusion-associated transmission have also been reported [9,11]. International studies have documented rising syphilis prevalence among blood donors in several developed countries [12,13]. In Canada, the incidence of syphilis among blood donors increased over time, and a retrospective study from China also demonstrated rising infection rates linked to high-risk sexual behavior and population mobility [14,15]. In Saudi Arabia, the reported prevalence of syphilis among blood donors remains relatively low compared with international reports; however, regional studies have shown variations in seropositivity rates across the country [16,17,18,19]. A nationwide study conducted in 2020 further identified syphilis as one of the major TTIs among Saudi blood donors [5].

HTLV-I was the first human retrovirus identified and is associated with adult T-cell leukemia and HTLV-associated myelopathy/tropical spastic paraparesis (HAM/TSP) [20,21]. HTLV-II shares similar genomic features but is less commonly associated with clinical disease [22,23]. HTLV-I/II transmission occurs through sexual contact, breastfeeding, organ transplantation, and blood transfusion [24]. Globally, HTLV infection remains endemic in regions including sub-Saharan Africa, the Caribbean, South America, the Middle East, and southwestern Japan [21]. Although most infected individuals remain asymptomatic, HTLV infection is a major transfusion safety concern because infected donors can unknowingly transmit the virus [25,26]. In Saudi Arabia, national blood screening policies include routine HTLV-I/II testing [1]. Previous Saudi studies have reported low seroprevalence among blood donors, including studies conducted at King Khalid General Hospital, Buraidah Central Hospital Blood Bank, and King Faisal Specialist Hospital and Research Center [16,17,18].

Malaria remains a major global public health concern, with more than 600,000 malaria-related deaths reported annually [27]. The disease is caused by Plasmodium species transmitted primarily by infected female Anopheles mosquitoes, including *P. falciparum* and *P. vivax* [28,29]. In addition to vector-borne transmission, malaria may be transmitted through blood transfusion, organ transplantation, needlestick injuries, and congenital exposure [30,31]. Although the risk of transfusion-transmitted malaria is relatively low in non-endemic countries, asymptomatic donors with low parasitemia may evade routine screening and contribute to transmission [31,32]. Saudi Arabia has made substantial progress toward malaria elimination through long-standing national control programs; however, malaria remains endemic in the southwestern region bordering Yemen [33,34]. The central region hosts a large expatriate population from malaria-endemic regions, highlighting the importance of continued donor screening. Previous Saudi studies have reported low malaria seropositivity rates among blood donors, with some centers reporting no positive donor samples [18,19].

Despite ongoing national screening efforts, regional epidemiological data from large blood banks in the central region remains limited. Most published Saudi studies have focused on individual institutions or single TTIs. Therefore, this study assessed the prevalence of syphilis, HTLV-I/II, and malaria among blood donors at the Riyadh Regional Laboratory and Blood Bank over a five-year period (2018–2022). In addition, the study described donor demographics, including age, sex, nationality, and ABO/RhD blood group distribution, and examined associations between donor characteristics and TTI seropositivity.

## 2. Materials and Methods

### 2.1. Study Design and Setting

This retrospective, observational study was conducted at the Riyadh Regional Laboratory and Blood Bank, which serves multiple tertiary hospitals in the central region of Saudi Arabia. The study period encompassed all blood donations received from 1 January 2018 to 31 December 2022.

### 2.2. Study Population

Data from all blood donors at the regional blood bank between 2018 and 2022 were collected. The target population was defined according to the standard national criteria: age between 18 and 65 years, body weight above 50 kg, hemoglobin of at least 12.5 g/dL for females and 13.5 g/dL for males, blood pressure within the accepted range (systolic 120–129 mmHg, diastolic 80–89 mmHg), pulse rate 60–100/min, normal body temperature, and no history of risk factors for transfusion-transmitted infections (TTIs) or recent travel to malaria-endemic regions [5,6].

### 2.3. Data Collection

Variables extracted from the blood bank database for each donation included age, gender, nationality, ABO blood group, RhD status, year of donation, and serological results for malaria, HTLV-I/II, and syphilis. Each record in the database represented a single blood donation episode, and unique donor identifiers were not available in the retrospective dataset to allow for the deduplication of repeat donors. Therefore, analyses were conducted at the donation level. Records with missing information for a given variable were excluded only from the corresponding sub-analysis. The proportion of missing data was minimal for most variables: age (0.3%), gender (0.0%), nationality (0.0%), ABO blood group (0.1%), RhD status (0.1%), and year of donation (0.0%). No records were missing serological results for the three TTIs, as these were the primary outcomes of interest.

Screening for TTIs was performed using standardized protocols at the Riyadh Regional Laboratory and Blood Bank. Malaria screening was conducted using an enzyme-linked immunosorbent assay (ELISA) (Bio-Rad Laboratories, Inc., Hercules, CA, USA) and positive-reactive samples were confirmed by microscopic examination of thick and thin blood films, following the Saudi Ministry of Health guidelines. Anti-HTLV-I/II antibodies were screened using chemiluminescent assays (CIAs) (Alinity S Abbott Laboratories, Chicago, IL, USA). Syphilis screening was performed using treponemal-specific ELISA or CIAs for detection of anti-*Treponema pallidum* antibodies (ETI-Max 3000 DiaSorin S.p.A., Saluggia, Italy). Reactive screening samples for syphilis and HTLV I/II were subjected to confirmatory testing using Western blot (for HTLV) or *Treponema pallidum* particle agglutination (TPPA) assays; however, confirmatory testing protocols were not uniformly documented across all five years, and for the purposes of this analysis, reactivity on initial screening tests was considered as a positive result. This methodological limitation is addressed further in Section 6.

### 2.4. Ethical Approval

The study was approved by the Institutional Review Board (IRB) of King Saud Medical City in the central region of Saudi Arabia (Reference: H1R1-30-Apr23-01). The IRB waived the requirement for individual informed consent because all data were drawn from anonymous records that contained no identifiers.

### 2.5. Statistical Analysis

Data from 1 January 2018 to 31 December 2022 were exported to Microsoft Excel (Microsoft, Redmond, WA, USA) and analyzed using SPSS version 28 (IBM, Armonk, NY, USA). Categorical variables were summarized as counts and percentages. The Chi-square test was used to compare proportions across groups, and Fisher’s exact test was applied when expected cell counts were less than five. Multivariable logistic regression was performed for each TTI to assess independent associations with age group, gender, nationality, and year of donation, with adjusted odds ratios (aOR) and 95% confidence intervals (CI) reported. Continuous variables were summarized using means and standard deviations where appropriate. A *p*-value below 0.05 was considered statistically significant. The Cochran–Armitage test for trend was used to assess temporal trends in syphilis prevalence, and Poisson regression was performed to estimate the annual rate of change.

## 3. Results

### 3.1. Characteristics of the Blood Donors

A total of 217,543 blood donations were recorded over the study period, with an annual mean of 43,508 donations. The highest annual count was in 2022 (*n* = 46,122), and the lowest in 2020 (*n* = 40,331). The majority of donors were male (212,072; 97%), and 5471 (3%) were female. Saudi nationals contributed 104,453 donations (48%), while non-Saudi donors contributed 113,090 (52%). The most common ABO blood group was O (47%), followed by A (29%), B (19%), and AB (5%). RhD-positive donors made up 197,198 (91%) of the cohort, while 20,345 (9%) were RhD-negative (Table 1).

### 3.2. Distribution of Malaria, Anti-HTLV-I/ii, and Syphilis Antibody Positivity Between 2018 and 2022

The distribution of malaria, anti-HTLV-I/II, and syphilis antibody positivity was studied over a five-year period. All 58 malaria-positive cases (0.03%) were recorded in 2018, with no positive cases identified between 2019 and 2022. Anti-HTLV-I/II antibodies were detected in 464 donations (0.21%), with the highest annual proportion in 2019 (*n* = 184; 39.7%) and the lowest in 2022 (*n* = 32; 6.9%). Syphilis antibodies were detected in 1143 donations (0.52%), increasing from 124 cases in 2018 (10.8%) to 299 cases in 2022 (26.2%). A statistically significant association was observed between the year of donation and positivity for malaria (*p* < 0.0001), anti-HTLV-I/II (*p* < 0.0001), and syphilis antibodies (*p* < 0.0001) (Table 2 and Table 3).

### 3.3. Distribution of Malaria, Anti-HTLV-I/II, and Syphilis Antibody Positivity by Age, Gender, and Nationality

Positive cases were observed based on age, gender, and nationality. Malaria was most prevalent in the 31–45 year age group (*n* = 30; 51.7%), followed by the 46–65 group (*n* = 25; 43.1%), and the 18–30 group (*n* = 3; 5.2%), with a statistically significant association between age and malaria positivity (*p* < 0.0001). All 58 positive cases were detected in male donors, with no positive cases among female donors (*p* = 0.221). Malaria positivity was confined entirely to non-Saudi donors (*p* < 0.0001).

Among anti-HTLV-I/II positive cases, the highest proportion was in the 31–45 age group (*n* = 255; 55.0%), followed by the 18–30 group (*n* = 127; 27.4%), and the 46–65 group (*n* = 82; 17.7%). The association between age and anti-HTLV-I/II positivity was statistically significant (*p* = 0.016). Male donors accounted for 445 (95.9%) positive cases, while female donors for 19 (4.1%), with a statistically significant association between gender and anti-HTLV-I/II positivity (*p* = 0.030). Positive cases were distributed between non-Saudi donors (*n* = 258; 55.6%) and Saudi donors (*n* = 206; 44.4%), with no statistically significant association between nationality and anti-HTLV-I/II positivity (*p* = 0.118).

In addition, the highest proportion of positive cases with syphilis antibodies was observed in the 31–45 age group (*n* = 587; 51.4%), followed by the 18–30 group (*n* = 305; 26.7%), and the 46–65 group (*n* = 251; 22.0%). There was a statistically significant association between age and syphilis positivity (*p* < 0.0001). Positive cases of male donors were 1116 (97.6%) and female donors were 27 (2.4%); the association between gender and syphilis positivity was not statistically significant (*p* = 0.741). Non-Saudi donors contributed 707 positive cases (61.9%), and Saudi donors 436 (38.1%), with a statistically significant association between nationality and syphilis positivity (*p* < 0.0001) (Table 4).

Within-group percentages showed that malaria positivity was confined to male donors (100%) and non-Saudi donors (100%), and was highest in the 31–45 age group (51.7%). Anti-HTLV-I/II positivity was predominantly identified in male donors (95.9%), peaked in the 31–45 age group (55%), and was more frequent in non-Saudi donors (55.6%) than Saudi donors (44.4%). Syphilis antibody positivity was likewise highest in male donors (97.6%) and in the 31–45 age group (51.4%), with a substantially higher share in non-Saudi (61.9%) compared with Saudi donors (38.1%) (Table 5).

### 3.4. Distribution of Malaria, Anti-HTLV-I/ii, and Syphilis Antibody Positivity by ABO and RhD Blood Groups

The highest number of positive malaria cases was observed in the O-positive group (*n* = 24; 41.4%), followed by A-positive (*n* = 16; 27.6%), and B-positive (*n* = 13; 22.4%). The remaining positive cases were distributed across A-negative (*n* = 1), AB-positive (*n* = 1), B-negative (*n* = 2), and O-negative (*n* = 1). No statistically significant association was observed between the ABO/RhD blood group and malaria positivity (*p* = 0.698).

The highest proportion of anti-HTLV-I/II positive cases occurred in the O-positive group (*n* = 186; 40.1%), followed by A-positive (*n* = 121; 26.1%), and B-positive (*n* = 100; 21.6%). No statistically significant association was observed between the ABO/RhD blood group and anti-HTLV-I/II positivity (*p* = 0.214).

Similarly, the highest proportion of syphilis antibody-positive cases was noted in the O-positive group (*n* = 483; 42.3%), followed by A-positive (*n* = 298; 26.1%), and B-positive (*n* = 202; 17.7%). No statistically significant association was observed between the ABO/RhD blood group and syphilis positivity (*p* = 0.686) (Table 6 and Table 7).

### 3.5. Multivariable Logistic Regression Analysis

Multivariable logistic regression was performed to assess independent associations between donor characteristics and TTI positivity (Table 8). For malaria, the model could not be estimated due to complete separation, as all 58 cases occurred in non-Saudi male donors in 2018. For HTLV I/II, after adjusting for other factors, age remained significantly associated with positivity, with donors aged 31–45 (aOR 1.58, 95% CI 1.27–1.96) and 46–65 (aOR 1.87, 95% CI 1.42–2.47) having higher odds compared with the 18–30 age group. Donation year was also independently associated, with 2019 showing the highest odds compared with 2018 (aOR 1.72, 95% CI 1.36–2.19), and subsequent years showing lower odds. Gender and nationality were not independently associated with HTLV I/II positivity after adjustment.

For syphilis, age remained a significant independent predictor, with donors aged 31–45 (aOR 1.61, 95% CI 1.39–1.86) and 46–65 (aOR 2.40, 95% CI 2.03–2.84) having higher odds compared with the 18–30 group. Non-Saudi nationality was independently associated with higher odds of syphilis positivity (aOR 1.48, 95% CI 1.31–1.67). Compared with 2018, all subsequent years showed higher odds of syphilis positivity, with 2022 showing the highest (aOR 2.26, 95% CI 1.83–2.80). Gender was not independently associated with syphilis positivity.

## 4. Discussion

This study examined five years of donation records (2018–2022) from the Riyadh Regional Laboratory and Blood Bank to determine the prevalence of malaria, HTLV I/II, and syphilis among blood donors and to explore associations with donor demographics and ABO/RhD blood group. All three infections were relatively uncommon: malaria was almost absent after 2018; HTLV I/II remained at low prevalence throughout the study period; and syphilis was the most frequent infection and the only one to increase steadily over the study period.

The study recorded approximately 40,000 donations per year at the Riyadh Regional Blood Bank, which is higher than the volumes reported from lower-throughput regional centers in Saudi Arabia, such as Jazan and Hail [19,35]. However, this figure is relatively low compared with international centers in higher-income settings, where annual collections often exceed 70,000 [2]. As the capital and most populous city in the country, the central region would be expected to generate a much larger donor pool, and the comparatively modest turnout suggests opportunities for further initiatives to encourage voluntary blood donation [36].

A notable dip in donations was observed in 2020, when annual collections fell from 43,825 in 2019 to 40,331, representing a 7.9% decrease. This decline coincided with the onset of the COVID-19 pandemic in Saudi Arabia. The first confirmed case in the Kingdom was reported on 2 March 2020, followed by the implementation of nationwide curfews, suspension of international travel, and restrictions on non-essential activities [37]. These measures, together with concerns about infection and the disruption of community-based blood donation campaigns, affected blood donor attendance and blood collection services. Studies from Saudi Arabia reported substantial reductions in donor attendance and blood supply during the early phase of the pandemic [38,39]. Similar patterns of reduced blood donation during the pandemic have been reported globally, including in Canada, the United States, and other Gulf Cooperation Council countries, reflecting challenges related to lockdowns, fear of infection, reduced mobility, and diversion of healthcare resources [40,41,42]. The 2020 decline highlights the vulnerability of blood supply systems to public health emergencies and underscores the importance of maintaining robust donor recruitment and retention strategies during crises.

Males accounted for the majority of donors (97%), a pattern consistently reported across Saudi centers [3,16] and the wider region [43,44]. The very low proportion of female donors (3%) has also been documented elsewhere in Saudi Arabia and is commonly attributed to cultural beliefs, fear of needles, the perception that donation causes a harmful loss of blood, and frequent deferrals for low hemoglobin, menstruation, and pregnancy [4,6]. The predominance of male donors may reflect differences in awareness, accessibility, or eligibility [36]. Although males were the primary donors, the low participation of females underscores the importance of sustained educational campaigns that address these misconceptions and raise awareness of the minimal risks associated with donation [6].

When sex-stratified prevalence rates were calculated, female donors had a higher per capita prevalence of HTLV I/II (0.35%) compared with male donors (0.21%), despite the much smaller female denominator. For syphilis, the per capita prevalence was similar between females (0.49%) and males (0.53%). The apparent predominance of male positive cases for all three infections reflects the male-dominated donor pool rather than a higher biological susceptibility in males. After adjustment in multivariable logistic regression, gender was not independently associated with HTLV I/II or syphilis positivity.

When age-specific prevalence rates were calculated, the 46–65 age group showed the highest per capita prevalence for all three TTIs: 0.085% for malaria, 0.28% for HTLV I/II, and 0.85% for syphilis. While the 31–45 age group contributed the largest absolute number of cases, this reflects its predominance in the donor pool (55.1%) rather than the highest per capita risk.

Non-Saudi donors accounted for slightly more than half of the cohort (52%), a higher proportion than reported in the western region [19] and in the national dataset [3]. This aligns with the capital’s large expatriate workforce, underscoring the city’s role as the country’s administrative and economic center. Group O was the most common blood type (47%), and AB the least common (5%). Most donors were RhD-positive (91%), a distribution that closely matches other Saudi and regional reports [18,45].

Malaria is transmitted primarily by the bite of the female Anopheles mosquito, but can also be acquired through transfusion from asymptomatic, low-parasitemic donors [30,31]. Saudi Arabia has advanced toward elimination through sustained national control programs, although residual transmission persists in the southwestern regions bordering Yemen [33,34]. In the present cohort, malaria prevalence was extremely low (0.03%), with all positive cases confined to 2018 and none detected thereafter (Table 2). This pattern aligns with the disease’s near-elimination status nationally and with the arid, non-endemic environment [33]. The abrupt disappearance of malaria cases after 2018 warrants careful interpretation. While this pattern is consistent with Saudi Arabia’s progress toward malaria elimination, it may also reflect other factors, including changes in donor eligibility criteria, modifications in screening protocols or test kits, or shifts in the demographic composition of donors. In the absence of detailed records on screening algorithm changes and donor travel histories, we cannot definitively attribute the absence of cases after 2018 solely to the national elimination program. Nevertheless, the finding aligns with the country’s near-elimination status and the arid, non-endemic environment of the central region. All malaria-positive donors were male and non-Saudi, and most were 31–45 years old, consistent with exposure in endemic regions [30,46]. No association was found with the ABO or RhD groups, consistent with evidence that the red cell antigen profile does not meaningfully influence transfusion-related malaria risk in this setting [7,18].

HTLV I/II is transmitted through sexual contact, breastfeeding, and blood transfusion, and remains endemic in parts of Africa, South America, and Japan [24,47]. In this study, antibodies were detected in 0.21% of donations, comparable to figures from other low-endemic populations [47,48]. Positivity peaked in 2019 and declined in subsequent years (Table 2); whether this reflects a true epidemiological change or a difference in donor characteristics remains unclear. The decline in HTLV I/II positivity after the 2019 peak is notable and may be related to several factors, including changes in donor demographic composition during the pandemic. The COVID-19 pandemic led to significant disruptions in international travel and migration patterns, potentially reducing the proportion of donors originating from HTLV-endemic regions. Additionally, pandemic-related restrictions may have altered the demographic profile of blood donors, with a higher proportion of regular, lower-risk donors contributing during this period. However, as donor country of origin was not available in our dataset, we cannot confirm this hypothesis. It is also possible that the 2019 peak represented a temporal cluster that reverted to baseline, and longer-term surveillance will be needed to determine whether the decline is sustained. Most positive donors were male and aged 31–45, consistent with cumulative lifetime exposure, although Brazilian data suggest that female donors may have a higher per capita prevalence once the male-skewed denominator is taken into account [49]. No significant difference was observed between Saudi and non-Saudi donors, and no association with blood group was identified.

Syphilis, caused by *Treponema pallidum*, is transmitted predominantly through sexual contact and, less frequently, through transfusion [9,10]. It was the most common infection in this cohort (0.52%) and the only one to show a significant increasing trend over the study period (Cochran–Armitage trend test *p* < 0.001; Poisson regression IRR 1.21, 95% CI 1.15–1.28, *p* < 0.001). While the absolute number of positive cases more than doubled from 124 in 2018 to 299 in 2022, this increase was only partially explained by the higher donation volume in 2022 (46,122 vs. 41,949 in 2018), as the prevalence rate also increased from 0.30% to 0.65% over the same period. Comparable upward trends among blood donors have been reported in China, Canada, and Brazil, where rising seropositivity has been attributed to changing sexual behavior and delayed diagnosis [11,13,14]. The increasing prevalence also highlights the importance of effective treatment strategies, with recent studies exploring enhanced antibiotic regimens for both early and late syphilis to address potential treatment challenges [50]. In multivariable logistic regression, non-Saudi nationality was independently associated with higher odds of syphilis positivity (aOR 1.48, 95% CI 1.31–1.67), and the 46–65 age group showed the highest odds compared with the 18–30 group (aOR 2.40, 95% CI 2.03–2.84). Positivity was highest in the 31–45 age group by absolute counts, but age-specific prevalence rates were highest in the 46–65 age group (0.85%). The sustained rise in syphilis positivity from 2019 to 2022, observed even during the COVID-19 pandemic, warrants the careful consideration of pandemic-related factors. The pandemic may have influenced syphilis transmission and case detection in several ways. Reduced access to routine sexual health services and screening programs during lockdowns could have led to delayed diagnosis and treatment, potentially allowing for ongoing transmission. Changes in sexual behavior, including reduced casual partnerships during lockdowns followed by potential risk compensation as restrictions eased, may have influenced transmission patterns. Additionally, the diversion of healthcare resources toward COVID-19 response may have affected the completeness of screening and reporting. However, the consistent upward trend observed throughout the pandemic period suggests that the underlying increase in syphilis prevalence is a longer-term phenomenon that may predate and continue beyond the pandemic.

The finding that syphilis positivity was significantly higher among non-Saudi donors (aOR 1.48, 95% CI 1.31–1.67) underscores the need for more detailed demographic data collection. The non-Saudi category, as used in this study, encompasses a highly heterogeneous population originating from countries with markedly different epidemiological profiles. Without information on specific country of origin, duration of residence in Saudi Arabia, travel history, or socioeconomic characteristics, it is not possible to determine whether infections were acquired in the country of origin, during travel, or locally in Saudi Arabia. This distinction has important implications for screening policy and donor counseling. For example, donors from countries with high syphilis prevalence may benefit from enhanced pre-donation education and post-donation referral services, regardless of their duration of residence. Conversely, infections acquired locally would suggest the need for broader public health interventions. Future studies should incorporate more detailed demographic and epidemiological data to enable targeted, evidence-based risk stratification.

A critical methodological consideration is that our prevalence estimates for syphilis and HTLV I/II represent screening reactivity rather than confirmed infection. Screening assays may yield false-positive results, and without uniformly documented confirmatory testing, the true prevalence of active syphilis or HTLV infection may be lower than reported. For syphilis, reactive screening tests can occur in individuals with past treated infection, latent infection, or biological false positives. Therefore, the observed prevalence of 0.52% reflects the proportion of donors with reactive screening tests rather than the prevalence of active, transmissible syphilis. This distinction is important for clinical interpretation and should be considered when comparing our findings with studies that employed different testing algorithms.

No significant association was observed between the ABO or RhD groups and any of the three infections in univariate analyses or after adjustment for potential confounders in multivariable logistic regression. Although group O had the largest absolute number of positive cases for each pathogen, this reflects its predominance in the donor pool rather than any biological susceptibility. Some authors have proposed links between ABO antigens and susceptibility to specific pathogens, especially malaria [7], but the present data do not support a clinically meaningful effect, consistent with earlier Saudi work [16,18]. RhD-negative donors, who are often in shorter supply, showed no difference in infection rates compared with RhD-positive donors. Taken together, these findings indicate that, in this population, TTI risk is unlikely to be explained by ABO/RhD phenotype.

## 5. Conclusions

Among more than 217,000 donations collected at the Riyadh Regional Laboratory and Blood Bank from 2018 to 2022, we found a low overall burden of malaria (0.03%), HTLV I/II (0.21%), and syphilis (0.52%). Malaria positivity declined to zero after 2018, which is consistent with the success of Saudi Arabia’s national elimination program, although alternative explanations including changes in screening protocols and donor demographics cannot be excluded based on our data. HTLV I/II prevalence remained low, with no concerning trends. The most important finding is the steady rise in syphilis among donors over the five-year period, with non-Saudi donors aged 46–65 years showing the highest per capita prevalence. The clustering of malaria positivity among non-Saudi donors also indicates where future screening efforts should focus. The higher syphilis positivity among non-Saudi donors may reflect infections acquired in countries of origin, during travel, or locally; distinguishing between these scenarios requires more detailed data collection on country of origin, duration of residence, and travel history. ABO and RhD blood groups did not predict TTI status in this cohort in either univariate or multivariable analyses. The descriptive donor data, including the 47% O group share and the very high proportion of non-Saudi donors, provide a baseline for the central region’s blood services to plan inventory and emergency supply. Continued monitoring of TTI trends, periodic review of donor questionnaires, incorporation of unique donor identifiers to enable individual-level analyses, and prospective multicenter studies that capture detailed donor origin and longitudinal follow-up will be needed to further refine national screening policy.

## 6. Limitations

This study has several limitations that should be considered. First, the retrospective database lacked unique donor identifiers, preventing the deduplication of repeat donors across the study period. Consequently, our prevalence estimates represent donation-level prevalence rather than individual-level prevalence, and the statistical tests assume independence at the donation level, which may be violated if the same individual donated multiple times. Future prospective studies should incorporate unique donor identifiers to enable individual-level analyses. Second, as with any retrospective record review, the analysis depended on the completeness and accuracy of the original records, and donors with incomplete data for a given variable were excluded from the relevant sub-analysis. The proportion of missing data was minimal for most variables, however. Third, the study draws on a single, although large, blood bank, so the findings may not generalize directly to other Saudi regions with different donor profiles. Fourth, splitting nationality into ‘Saudi’ and ‘non-Saudi’ was driven by data availability, but it conceals what is in fact a very mixed expatriate population, and finer subgrouping would have allowed more useful risk stratification. Fifth, confirmatory testing protocols for reactive samples were not uniformly documented across all five years, which could affect the precision of the prevalence estimates, as our findings represent screening reactivity rather than confirmed infection. Sixth, donor follow-up data were not available, so incidence and reinfection patterns could not be examined. Seventh, the abrupt disappearance of malaria cases after 2018 could reflect changes in donor eligibility criteria, screening protocols, or testing methodologies rather than solely the success of the national elimination program; we have acknowledged this in our interpretation.

## Figures and Tables

**Table 1:** Study sample characteristics of blood donors from 2018 to 2022 at the Riyadh Regional Laboratory and Blood Bank (*n* = 217,543).

Variable	Option	N	%
Year	2018	41,949	19%
	2019	43,825	20%
	2020	40,331	19%
	2021	45,316	21%
	2022	46,122	21%
	Total	217,543	100%
Gender	Female	5471	3%
	Male	212,072	97%
	Total	217,543	100%
Nationality	Saudi	104,453	48%
	Non-Saudi	113,090	52%
	Total	217,543	100%
ABO	O	103,115	47%
	A	62,003	29%
	B	41,495	19%
	AB	10,930	5%
	Total	217,543	100%
RhD	Negative	20,345	9%
	Positive	197,198	91%
	Total	217,543	100%

**Table 2:** Prevalence rates of malaria, anti-HTLVI/II and syphilis.

Year	Malaria		Anti-HTLV I/II		Syphilis	
	Positive	Prevalence (95% CI)	Positive	Prevalence (95% CI)	Positive	Prevalence (95% CI)
2018	58	0.14% (0.11–0.18)	110	0.26% (0.22–0.32)	124	0.30% (0.25–0.35)
2019	0	0.00% (0.00–0.01)	184	0.42% (0.36–0.48)	259	0.59% (0.52–0.67)
2020	0	0.00% (0.00–0.01)	61	0.15% (0.12–0.19)	231	0.57% (0.50–0.65)
2021	0	0.00% (0.00–0.01)	77	0.17% (0.13–0.21)	230	0.51% (0.45–0.58)
2022	0	0.00% (0.00–0.01)	32	0.07% (0.05–0.10)	299	0.65% (0.58–0.72)

For syphilis, the Cochran–Armitage test for trend showed a significantly increasing trend over the study period (*p* < 0.001). Poisson regression confirmed a significant annual increase in syphilis prevalence (Incidence Rate Ratio 1.21, 95% CI 1.15–1.28, *p* < 0.001).

**Table 3:** Percentage of positive cases for malaria, anti-HTLV-I/II, and syphilis antibody across the study period (2018–2022) (*n* = 217,543).

Year		Malaria	Anti-HTLV I/II	Syphilis Antibody
		+ve	+ve	+ve
2018	Count	58	110	124
	% within	0.03%	23.7%	10.8%
2019	Count	0	184	259
	% within	0.0%	39.7%	22.7%
2020	Count	0	61	231
	% within	0.0%	13.1%	20.2%
2021	Count	0	77	230
	% within	0.0%	16.6%	20.1%
2022	Count	0	32	299
	% within	0.0%	6.9%	26.2%
Total	Count	58	464	1143
	% within	100%	100%	100%

**Table 4:** Distribution of malaria, anti-HTLV-I/II, and syphilis antibody positivity by age, gender, and nationality (*n* = 217,543).

			Malaria			Anti-HTLV I/II			Syphilis Antibody		
			−ve	+ve	Total	−ve	+ve	Total	−ve	+ve	Total
Gender	Male	Count	212,014	58	212,072	211,627	445	212,072	210,956	1116	212,072
		% within	97.5%	100%	97.5%	97.5%	95.9%	97.5%	97.5%	97.6%	97.5%
	Female	Count	5471	0	5471	5452	19	5471	5444	27	5471
		% within	2.5%	0.0%	2.5%	2.5%	4.1%	2.5%	2.5%	2.4%	2.5%
	Total	Count	217,485	58	217,543	217,079	464	217,543	216,400	1143	217,543
		% within	100%	100%	100%	100%	100%	100%	100%	100%	100%
		*p*-value	0.221			0.030			0.741		
Age	18–30	Count	68,337	3	68,340	68,213	127	68,340	68,035	305	68,340
		% within	31.4%	5.2%	31.4%	31.4%	27.4%	31.4%	31.4%	26.7%	31.4%
	31–45	Count	119,731	30	119,761	119,506	255	119,761	119,174	587	119,761
		% within	55.1%	51.7%	55.1%	55.1%	55%	55.1%	55.1%	51.4%	55.1%
	46–65	Count	29,417	25	29,442	29,360	82	29,442	29,191	251	29,442
		% within	13.5%	43.1%	13.5%	13.5%	17.7%	13.5%	13.5%	22.0%	13.5%
	Total	Count	217,485	58	217,543	217,079	464	217,543	216,400	1143	217,543
		% within	100%	100%	100%	100%	100%	100%	100.0%	100.0%	100.0%
		*p*-value		0.0001			0.016			0.0001	
Nationality	Saudi	Count	104,453	0	104,453	104,247	206	104,453	104,017	436	104,453
		% within	48%	0.0%	48%	48%	44.4%	48%	48.1%	38.1%	48.0%
	Non-Saudi	Count	113,032	58	113,090	112,832	258	113,090	112,383	707	113,090
		% within	52%	100%	52%	52%	55.6%	52%	51.9%	61.9%	52.0%
	Total	Count	217,485	58	217,543	217,079	464	217,543	216,400	1143	217,543
		% within	100%	100%	100%	100%	100%	100%	100%	100%	100%
		*p*		0.0001			0.118			0.0001	

**Table 5:** Percentage of positive cases for malaria, anti-HTLV-I/II, and syphilis antibody by age, gender, and nationality (*n* = 217,543).

			Malaria	Anti-HTLV I/II	Syphilis Antibody
			+ve	+ve	+ve
Gender	Male	Count	58	445	1116
		% within	100%	95.9%	97.6%
	Female	Count	0	19	27
		% within	0.0%	4.1%	2.4%
	Total	Count	58	464	1143
		% within	100%	100%	100%
Age	18–30	Count	3	127	305
		% within	5.2%	27.4%	26.7%
	31–45	Count	30	255	587
		% within	51.7%	55%	51.4%
	46–65	Count	25	82	251
		% within	43.1%	17.7%	22.0%
	Total	Count	58	464	1143
		% within	100%	100%	100.0%
Nationality	Saudi	Count	0	206	436
		% within	0.0%	44.4%	38.1%
	Non-Saudi	Count	58	258	707
		% within	100%	55.6%	61.9%
	Total	Count	58	464	1143
		% within	100%	100%	100%

**Table 6:** Distribution of malaria, anti-HTLV-I/II, and syphilis antibody positivity by ABO and RhD blood group (*n* = 217,543).

			Malaria			Anti-HTLV I/II			Syphilis Antibody		
			−ve	+ve	Total	−ve	+ve	Total	−ve	+ve	Total
Blood Group	A −ve	Count	5571	1	5572	5559	13	5572	5548	24	5572
		% within	2.6%	1.7%	2.6%	2.6%	2.8%	2.6%	2.6%	2.1%	2.6%
	A +ve	Count	56,415	16	56,431	56,310	121	56,431	56,133	298	56,431
		% within	25.9%	27.6%	25.9%	25.9%	26.1%	25.9%	25.9%	26.1%	25.9%
	AB −ve	Count	934	0	934	932	2	934	927	7	934
		% within	0.4%	0.0%	0.4%	0.4%	0.4%	0.4%	0.4%	0.6%	0.4%
	AB +ve	Count	9995	1	9996	9982	14	9996	9934	62	9996
		% within	4.6%	1.7%	4.6%	4.6%	3.0%	4.6%	4.6%	5.4%	4.6%
	B −ve	Count	3498	2	3500	3496	4	3500	3480	20	3500
		% within	1.6%	3.4%	1.6%	1.6%	0.9%	1.6%	1.6%	1.7%	1.6%
	B +ve	Count	37,982	13	37,995	37,895	100	37,995	37,793	202	37,995
		% within	17.5%	22.4%	17.5%	17.5%	21.6%	17.5%	17.5%	17.7%	17.5%
	O −ve	Count	10,338	1	10,339	10,315	24	10,339	10,292	47	10,339
		% within	4.8%	1.7%	4.8%	4.8%	5.2%	4.8%	4.8%	4.1%	4.8%
	O +ve	Count	92,752	24	92,776	92,590	186	92,776	92,293	483	92,776
		% within	42.6%	41.4%	42.6%	42.7%	40.1%	42.6%	42.6%	42.3%	42.6%
	Total	Count	217,485	58	217,543	217,079	464	217,543	216,400	1143	217,543
		% within	100%	100%	100%	100.0%	100.0%	100.0%	100%	100%	100%
		*p*	0.698			0.214			0.686		

**Table 7:** Percentage of positive cases for malaria, anti-HTLV-I/II, and syphilis antibody by ABO and RhD blood groups (*n* = 217,543).

			Malaria	Anti-HTLV I/II	Syphilis Antibody
			+ve	+ve	+ve
Blood Group	A −ve	Count	1	13	24
		% within	1.7%	2.8%	2.1%
	A +ve	Count	16	121	298
		% within	27.6%	26.1%	26.1%
	AB −ve	Count	0	2	7
		% within	0.0%	0.4%	0.6%
	AB +ve	Count	1	14	62
		% within	1.7%	3.0%	5.4%
	B −ve	Count	2	4	20
		% within	3.4%	0.9%	1.7%
	B +ve	Count	13	100	202
		% within	22.4%	21.6%	17.7%
	O −ve	Count	1	24	47
		% within	1.7%	5.2%	4.1%
	O +ve	Count	24	186	483
		% within	41.4%	40.1%	42.3%
	Total	Count	58	464	1143
		% within	100%	100.0%	100%

**Table 8:** Multivariable logistic regression analysis of factors associated with malaria, HTLV I/II, and syphilis positivity.

Variable	Category	Malaria aOR (95% CI)	*p*-Value	HTLV I/II aOR (95% CI)	*p*-Value	Syphilis aOR (95% CI)	*p*-Value
Age	18–30	Reference		Reference		Reference	
	31–45	3.10 (0.94–10.21)	0.062	1.58 (1.27–1.96)	<0.001	1.61 (1.39–1.86)	<0.001
	46–65	10.52 (3.18–34.78)	<0.001	1.87 (1.42–2.47)	<0.001	2.40 (2.03–2.84)	<0.001
Gender	Female	Reference		Reference		Reference	
	Male	— *	— *	0.79 (0.50–1.26)	0.326	0.93 (0.63–1.38)	0.721
Nationality	Saudi	Reference		Reference		Reference	
	Non-Saudi	— *	— *	1.13 (0.94–1.37)	0.192	1.48 (1.31–1.67)	<0.001
Year	2018	Reference		Reference		Reference	
	2019	— *	— *	1.72 (1.36–2.19)	<0.001	2.06 (1.66–2.55)	<0.001
	2020	— *	— *	0.58 (0.42–0.79)	0.001	1.92 (1.54–2.39)	<0.001
	2021	— *	— *	0.72 (0.53–0.96)	0.026	1.76 (1.41–2.20)	<0.001
	2022	— *	— *	0.30 (0.20–0.44)	<0.001	2.26 (1.83–2.80)	<0.001

* Malaria multivariable model could not be estimated due to complete separation (all cases in non-Saudi males in 2018).

## Data Availability

The original contributions presented in this study are included in the article. Further inquiries can be directed to the corresponding author.

## References

[1] Myers D.J., Collins R.A. (2026). Blood Donation. StatPearls.

[2] Kanagasabai U., Selenic D., Chevalier M.S., Drammeh B., Qualls M., Shiraishi R.W., Bock N., Benech I., Mili F.D. (2021). Evaluation of the WHO Global Database on Blood Safety. Vox Sang..

[3] Alsughayyir J., Almalki Y., Alalshaik M., Aljoni I., Kandel M., Alfhili M.A., Alabdullateef A. (2022). Demography and Blood Donation Trends in Saudi Arabia: A Nationwide Retrospective, Cross-Sectional Study. Saudi J. Biol. Sci..

[4] Davison T., Masser B., Gemelli C. (2019). Deferred and Deterred: A Review of Literature on the Impact of Deferrals on Blood Donors. ISBT Sci. Ser..

[5] Alsughayyir J., Almalki Y., Alburayk I., Alalshaik M., Aljoni I., Kandel M., Alfhili M.A., Alabdullateef A.A. (2022). Prevalence of Transfusion-Transmitted Infections in Saudi Arabia Blood Donors: A Nationwide, Cross-Sectional Study. Saudi Med. J..

[6] Blood Donor Selection: Guidelines on Assessing Donor Suitability for Blood Donation. https://www.who.int/publications/i/item/9789241548519.

[7] Cooling L. (2015). Blood Groups in Infection and Host Susceptibility. Clin. Microbiol. Rev..

[8] Sun Y., Wang L., Niu J., Ma T., Xing L., Song A., Wang W., Shen Y., Yang J. (2022). Distribution Characteristics of ABO Blood Groups in China. Heliyon.

[9] Stoltey J.E., Cohen S.E. (2015). Syphilis Transmission: A Review of the Current Evidence. Sex. Health.

[10] Giacani L., Brandt S.L., Ke W., Reid T.B., Molini B.J., Iverson-Cabral S., Ciccarese G., Drago F., Lukehart S.A., Centurion-Lara A. (2015). Transcription of TP0126, Treponema Pallidum Putative OmpW Homolog, Is Regulated by the Length of a Homopolymeric Guanosine Repeat. Infect. Immun..

[11] Tamrakar P., Bett C., Molano R.D., Ayub A., Asher D.M., Gregori L. (2021). Effect of Storage on Survival of Infectious Treponema Pallidum Spiked in Whole Blood and Platelets. Transfusion.

[12] Kluppel G.P.Z., de Oliveira J.B.F., Skare T.L., Favero K.B., Almeida P.T.R., Nisihara R.M. (2022). Seropositivity for Syphilis among Brazilian Blood Donors. A Retrospective Study 2015–2020. Transfus. Apher. Sci..

[13] Zheng X., Ding W., Ling X., Shi J., Dong J., Yu G., Chen Y., Li R., Xu L., Li X. (2022). Prevalence of Treponema Pallidum Antibody among Volunteer Blood Donors in China. Can. J. Infect. Dis. Med. Microbiol..

[14] O’Brien S., Drews S., Yi Q.-L., Osmond L., Tran V., Zhou H., Goldman M. (2023). Monitoring Syphilis Serology in Blood Donors: Is There Utility as a Surrogate Marker of Early Transfusion Transmissible Infection Behavioral Risk?. Transfusion.

[15] Chen X., Liu Q., Sun P., Yuan S., Liao H., Zhang X. (2022). Prevalence of Syphilis Infections Among Volunteer Blood Donors in Jinan Blood Center, China: A 15-Year Retrospective Study. Infect. Drug Resist..

[16] Alaidarous M., Choudhary R.K., Waly M.I., Mir S., Bin Dukhyil A., Banawas S.S., Alshehri B.M. (2018). The Prevalence of Transfusion-Transmitted Infections and Nucleic Acid Testing among Blood Donors in Majmaah, Saudi Arabia. J. Infect. Public Health.

[17] Elyamany G., Al amro M., Pereira W., Alsuhaibani O. (2016). Prevalence of Syphilis among Blood and Stem Cell Donors in Saudi Arabia: An Institutional Experience. Electron. Physician.

[18] Al Abdulmonem W., Shariq A., Alqossayir F., AbaAlkhail F., Al-Musallam A., Alzaaqi F., Aloqla A., Alodhaylah S., Alsugayyir A., Aldoubiab R. (2020). Sero-Prevalence ABO and Rh Blood Groups and Their Associated Transfusion-Transmissible Infections among Blood Donors in the Central Region of Saudi Arabia. J. Infect. Public Health.

[19] Altayar M., Jalal M., Kabrah A., Qashqari F., Jalal N., Faidah H., Baghdadi M., Kabrah S. (2022). Prevalence and Association of Transfusion Transmitted Infections with ABO and Rh Blood Groups among Blood Donors in the Western Region of Saudi Arabia: A 7-Year Retrospective Analysis. Medicina.

[20] Poiesz B.J., Ruscetti F.W., Gazdar A.F., Bunn P.A., Minna J.D., Gallo R.C. (1980). Detection and Isolation of Type C Retrovirus Particles from Fresh and Cultured Lymphocytes of a Patient with Cutaneous T-Cell Lymphoma. Proc. Natl. Acad. Sci. USA.

[21] Willems L., Hasegawa H., Accolla R., Bangham C., Bazarbachi A., Bertazzoni U., Carneiro-Proietti A.B.d.F., Cheng H., Chieco-Bianchi L., Ciminale V. (2017). Reducing the Global Burden of HTLV-1 Infection: An Agenda for Research and Action. Antivir. Res..

[22] Kalyanaraman V.S., Sarngadharan M.G., Robert-Guroff M., Miyoshi I., Blayney D., Golde D., Gallo R.C. (1982). A New Subtype of Human T-Cell Leukemia Virus (HTLV-II) Associated with a T-Cell Variant of Hairy Cell Leukemia. Science.

[23] Martinez M., Al-Saleem J., Green P. (2019). Comparative Virology of HTLV-1 and HTLV-2. Retrovirology.

[24] Kalinichenko S., Komkov D., Mazurov D. (2022). HIV-1 and HTLV-1 Transmission Modes: Mechanisms and Importance for Virus Spread. Viruses.

[25] Murphy E.L. (2024). HTLV-1 and Blood Donation. Br. J. Haematol..

[26] Rosadas C., Menezes M.L.B., Galvão-Castro B., Assone T., Miranda A.E., Aragón M.G., Caterino-de-Araujo A., Taylor G.P., Ishak R. (2021). Blocking HTLV-1/2 Silent Transmission in Brazil: Current Public Health Policies and Proposal for Additional Strategies. PLoS Negl. Trop. Dis..

[27] Monroe A., Williams N.A., Ogoma S., Karema C., Okumu F. (2022). Reflections on the 2021 World Malaria Report and the Future of Malaria Control. Malar. J..

[28] Meibalan E., Marti M. (2017). Biology of Malaria Transmission. Cold Spring Harb. Perspect. Med..

[29] Bousema T., Drakeley C. (2011). Epidemiology and Infectivity of *Plasmodium falciparum* and *Plasmodium vivax* Gametocytes in Relation to Malaria Control and Elimination. Clin. Microbiol. Rev..

[30] Ahmadpour E., Foroutan-Rad M., Majidiani H., Moghaddam S.M., Hatam-Nahavandi K., Hosseini S.-A., Rahimi M.T., Barac A., Rubino S., Zarean M. (2019). Transfusion-Transmitted Malaria: A Systematic Review and Meta-Analysis. Open Forum Infect. Dis..

[31] Niederhauser C., Galel S.A. (2022). Transfusion-Transmitted Malaria and Mitigation Strategies in Nonendemic Regions. Transfus. Med. Hemother..

[32] Lin H. (2021). Current Situation of Transfusion-Transmitted Malaria in China. J. Trop. Med..

[33] Al-Mekhlafi H.M., Madkhali A.M., Ghailan K.Y., Abdulhaq A.A., Ghzwani A.H., Zain K.A., Atroosh W.M., Alshabi A., Khadashi H.A., Darraj M.A. (2021). Residual Malaria in Jazan Region, Southwestern Saudi Arabia: The Situation, Challenges and Climatic Drivers of Autochthonous Malaria. Malar. J..

[34] Madkhali A.M., Ghzwani A.H., Al-Mekhlafi H.M. (2022). Comparison of Rapid Diagnostic Test, Microscopy, and Polymerase Chain Reaction for the Detection of *Plasmodium falciparum* Malaria in a Low-Transmission Area, Jazan Region, Southwestern Saudi Arabia. Diagnostics.

[35] Alshehri A.A., Adebayo Irekeola A., Merae Alshahrani M., Mohammed Abdul K.S., Ahmed Asiri S., Aboluluy B.F., Abdullah Al Awadh A., Hassan Alhasaniah A., Abdullah Almazni I., Alshamrani S.A. (2024). Serological Markers of Transfusion Transmissible Infections and ABO Blood Groups in Najran, Saudi Arabia. Saudi Med. J..

[36] Otifi H.M., Asiri M.A., Ahmad M.T., AlAsiri A.A.A., AlOudhah S.M., Alshorfi H.A., Alalmai A.M., Alam M.M. (2020). Measuring Public Awareness about Blood Donation in Assir, South-Western Saudi Arabia. Transfus. Clin. Biol..

[37] MOH News MOH Reports First Case of Coronavirus Infection. https://www.moh.gov.sa/en/ministry/mediacenter/news/pages/news-2020-03-02-002.aspx?utm_source=chatgpt.com.

[38] Yahia A.I.O. (2020). Management of Blood Supply and Demand during the COVID-19 Pandemic in King Abdullah Hospital, Bisha, Saudi Arabia. Transfus. Apher. Sci..

[39] Hakami N.Y., Al-Sulami A.J., Alhazmi W.A., Qadah T.H., Bawazir W.M., Hamadi A.Y., Owaidah A.Y., Alhefzi R.A., Hamdi F.Y., Maqnas A. (2022). Impact of COVID-19 on Blood Donation and Supply: A Multicenter Cross-Sectional Study from Saudi Arabia. Biomed. Res. Int..

[40] Prokopchuk-Gauk O., Petraszko T., Nahirniak S., Doncaster C., Levy I. (2021). Blood Shortages Planning in Canada: The National Emergency Blood Management Committee Experience during the First 6 Months of the COVID-19 Pandemic. Transfusion.

[41] Basavaraju S.V., Free R.J., Chavez Ortiz J.L., Stewart P., Berger J., Sapiano M.R.P. (2023). Impact of the COVID-19 Pandemic on Blood Donation and Transfusions in the United States in 2020. Transfusion.

[42] WHO EMRO COVID-19-Related Challenges in Blood Donation in the United Arab Emirates. https://www.emro.who.int/emhj-volume-30-2024/volume-30-issue-7/covid-19-related-challenges-in-blood-donation-in-the-united-arab-emirates.html?utm_source=chatgpt.com.

[43] Saba N., Nasir J.A., Waheed U., Aslam S., Mohammad I., Wazeer A., Ahmed S., Nisar M. (2021). Seroprevalence of Transfusion-Transmitted Infections among Voluntary and Replacement Blood Donors at the Peshawar Regional Blood Centre, Khyber Pakhtunkhwa, Pakistan. J. Lab. Physicians.

[44] Aabdien M., Selim N., Himatt S., Hmissi S., Merenkov Z., AlKubaisi N., Abdel-Rahman M.E., Abdelmola A., Khelfa S., Farag E. (2020). Prevalence and Trends of Transfusion Transmissible Infections among Blood Donors in the State of Qatar, 2013–2017. BMC Infect. Dis..

[45] AlShamlan N.A., Al Shammari M.A., AlOmar R.S., Gari D., AlAbdulKader A.M., Motabgani S., Farea A., Darwish M.A. (2021). ABO and Rhesus Blood Group Distribution and Blood Donation Willingness Among First-Year Health Students in a Saudi University. J. Blood Med..

[46] Fambirai T., Chimbari M.J., Ndarukwa P. (2022). Global Cross-Border Malaria Control Collaborative Initiatives: A Scoping Review. Int. J. Environ. Res. Public Health.

[47] Chang L., Ou S., Shan Z., Zhu F., Ji H., Rong X., Guo F., Jiang X., Sun H., Yan Y. (2021). Seroprevalence of Human T-Lymphotropic Virus Infection among Blood Donors in China: A First Nationwide Survey. Retrovirology.

[48] Rosadas C., Taylor G.P. (2022). HTLV-1 and Co-Infections. Front. Med..

[49] Miranda C., Utsch-Gonçalves D., Piassi F.C.C., Loureiro P., Gomes I., Ribeiro M.A., de Almeida-Neto C., Blatyta P., Amorim L., Garcia Mateos S.O. (2022). Prevalence and Risk Factors for Human T-Cell Lymphotropic Virus (HTLV) in Blood Donors in Brazil-A 10-Year Study (2007–2016). Front. Med..

[50] Drago F., Ciccarese G., Broccolo F., Sartoris G., Stura P., Esposito S., Rebora A., Parodi A. (2016). A New Enhanced Antibiotic Treatment for Early and Late Syphilis. J. Glob. Antimicrob. Resist..

